# Epidemiology of *Shigella* infections and diarrhea in the first two years of life using culture-independent diagnostics in 8 low-resource settings

**DOI:** 10.1371/journal.pntd.0008536

**Published:** 2020-08-17

**Authors:** Elizabeth T. Rogawski McQuade, Fariha Shaheen, Furqan Kabir, Arjumand Rizvi, James A. Platts-Mills, Fatima Aziz, Adil Kalam, Shahida Qureshi, Sarah Elwood, Jie Liu, Aldo A. M. Lima, Gagandeep Kang, Pascal Bessong, Amidou Samie, Rashidul Haque, Estomih R. Mduma, Margaret N. Kosek, Sanjaya Shrestha, Jose Paulo Leite, Ladaporn Bodhidatta, Nicola Page, Ireen Kiwelu, Sadia Shakoor, Ali Turab, Sajid Bashir Soofi, Tahmeed Ahmed, Eric R. Houpt, Zulfiqar Bhutta, Najeeha Talat Iqbal

**Affiliations:** 1 Department of Public Health Sciences, University of Virginia, Charlottesville, Virginia, United States of America; 2 Division of Infectious Diseases & International Health, University of Virginia, Charlottesville, Virginia, United States of America; 3 Department of Pediatrics and Child Health, Aga Khan University, Karachi, Pakistan; 4 Federal University of Ceara, Fortaleza, Brazil; 5 Christian Medical College, Vellore, India; 6 University of Venda, Thohoyandou, South Africa; 7 International Centre for Diarrheal Disease Research, Bangladesh, Dhaka, Bangladesh; 8 Haydom Global Health Research Centre, Haydom, Tanzania; 9 Asociación Benéfica PRISMA, Iquitos, Peru; 10 Walter Reed/AFRIMS Research Unit, Nepal, Kathmandu, Nepal; 11 Fundação Oswaldo Cruz (Fiocruz), Rio de Janeiro, Brazil; 12 Armed Forces Research Institute of Medical Sciences (AFRIMS), Bangkok, Thailand; 13 National Institute for Communicable Diseases, Johannesburg, South Africa; 14 Kilimanjaro Clinical Research Institute, Moshi, Tanzania; 15 Department of Pathology and Laboratory Medicine, Aga Khan University, Karachi, Pakistan; 16 Department of Pediatrics and Child Health and Biological & Biomedical Sciences, Aga Khan University, Karachi, Pakistan; Johns Hopkins University Bloomberg School of Public Health, UNITED STATES

## Abstract

Culture-independent diagnostics have revealed a larger burden of *Shigella* among children in low-resource settings than previously recognized. We further characterized the epidemiology of *Shigella* in the first two years of life in a multisite birth cohort. We tested 41,405 diarrheal and monthly non-diarrheal stools from 1,715 children for *Shigella* by quantitative PCR. To assess risk factors, clinical factors related to age and culture positivity, and associations with inflammatory biomarkers, we used log-binomial regression with generalized estimating equations. The prevalence of *Shigella* varied from 4.9%-17.8% in non-diarrheal stools across sites, and the incidence of *Shigella*-attributable diarrhea was 31.8 cases (95% CI: 29.6, 34.2) per 100 child-years. The sensitivity of culture compared to qPCR was 6.6% and increased to 27.8% in *Shigella*-attributable dysentery. *Shigella* diarrhea episodes were more likely to be severe and less likely to be culture positive in younger children. Older age (RR: 1.75, 95% CI: 1.70, 1.81 per 6-month increase in age), unimproved sanitation (RR: 1.15, 95% CI: 1.03, 1.29), low maternal education (<10 years, RR: 1.14, 95% CI: 1.03, 1.26), initiating complementary foods before 3 months (RR: 1.10, 95% CI: 1.01, 1.20), and malnutrition (RR: 0.91, 95% CI: 0.88, 0.95 per unit increase in weight-for-age z-score) were risk factors for *Shigella*. There was a linear dose-response between *Shigella* quantity and myeloperoxidase concentrations. The burden of *Shigella* varied widely across sites, but uniformly increased through the second year of life and was associated with intestinal inflammation. Culture missed most clinically relevant cases of severe diarrhea and dysentery.

## Author summary

*Shigella* is the second leading cause of diarrhea morbidity and mortality among children in low and middle-income countries. We characterized the epidemiology of *Shigella* using highly sensitive diagnostic methods in 41,405 diarrheal and monthly non-diarrheal stools from the first two years of life in a multisite birth cohort. The prevalence of *Shigella* varied from 4.9%-17.8% across sites, and the incidence of *Shigella*-attributable diarrhea was 31.8 cases (95% CI: 29.6, 34.2) per 100 child-years. *Shigella* diarrhea episodes were more likely to be severe and less likely to be culture positive in younger children. Older age, unimproved sanitation, low maternal education, initiating complementary foods before 3 months, and malnutrition were risk factors for *Shigella*. There was a linear dose-response between *Shigella* quantity and myeloperoxidase, a marker of intestinal inflammation, which suggests a potential mechanism for the impact of *Shigella* on child growth. Because culture missed most clinically relevant cases of severe diarrhea and dysentery, molecular diagnostics may be important tools in upcoming *Shigella* vaccine trials.

## Introduction

*Shigella* is the second leading cause of diarrhea morbidity and mortality among children in low and middle-income countries, accounting for approximately 60,000 deaths in 2016 [1]. An invasive Gram-negative rod, *Shigella* has a low infectious inoculum, and both fecal-oral and direct person-to-person transmission can occur [2]. *Shigella* is strongly associated with dysentery; correspondingly, the WHO guidelines recommend treatment of all pediatric cases of dysentery with ciprofloxacin or azithromycin for presumed *Shigella* infection [3].

The recent use of quantitative PCR for *Shigella* detection revealed a more than five times higher burden of *Shigella*-attributable diarrhea among children in low-resource settings than previously recognized using culture-based diagnostics [4–6]. Importantly, the majority of *Shigella* burden was associated with watery diarrhea, not dysentery [5]. A recent meta-analysis showed that the proportion of *Shigella* infections that present with dysentery has been decreasing, and that *Shigella* infections overall had a stronger association with mortality than *Shigella*-associated cases of dysentery [7]. Furthermore, even in the absence of diarrheal symptoms, *Shigella* has been associated with impaired linear growth [6,8,9]. WHO treatment guidelines do not currently recommend treatment for the majority of *Shigella* infections that may be associated with adverse outcomes, such that there may be missed treatment opportunities. Increasing rates of fluoroquinolone and macrolide resistance have highlighted the need for novel interventions, and particularly increased the urgency of the development of a *Shigella* vaccine [10], which may offer a more sustainable solution.

Given our recently revised understanding of the magnitude of *Shigella* disease burden and in preparation for *Shigella* vaccine trials, a better understanding of the epidemiology of *Shigella* infections among children in low-resource settings is needed. We describe the burden, diagnostic and clinical characteristics, risk factors, and seasonality of *Shigella* in the first two years of life in 8 low-resource settings.

## Methods

The MAL-ED study was conducted in eight sites: Dhaka (Bangladesh), Vellore (India), Bhaktapur (Nepal), Naushero Feroze (Pakistan), Venda (South Africa), Haydom (Tanzania), Fortaleza (Brazil), and Loreto (Peru), as previously described [11]. Briefly, between November 2009 and February 2012, children were recruited within 17 days of birth if maternal age was ≥ 16 years, their family intended to remain in the area for 6+ months, the child was a singleton pregnancy, birthweight was ≥ 1500 g, the child was not diagnosed with severe disease, and their siblings were not in the study. Fieldworkers conducted active surveillance for child illnesses, antibiotic use, and feeding practices twice weekly until two years of age. Anthropometry was measured monthly. Diarrheal stool samples were collected during diarrhea defined by maternal report of three or more loose stools in 24 hours or one stool with visible blood. Clinical characteristics, including blood observed in stool, were caregiver-reported. Severe diarrhea was defined using the CODA score, which has been previously validated against hospitalization [12,13] and is more appropriate for *Shigella* than the Vesikari score, which was validated for rotavirus. Non-diarrheal stool samples were collected monthly (at least 3 days distant to a diarrhea episode). Weight-for-age (WAZ) and length-for-age z-scores (LAZ) were calculated using 2006 WHO child growth standards [14]. Socioeconomic status (SES) was summarized using a construct of water, assets, maternal education, and income and was averaged over 4 biannual surveys [15].

### Ethics statement

The study was approved by the Institutional Review Board for Health Sciences Research, University of Virginia, USA as well as the respective governmental, local institutional, and collaborating institutional ethical review boards at each site: Ethical Review Committee, ICDDR,B (Bangladesh); Committee for Ethics in Research, Universidade Federal do Ceara; National Ethical Research Committee, Health Ministry, Council of National Health (Brazil); Institutional Review Board, Christian Medical College, Vellore; Health Ministry Screening Committee, Indian Council of Medical Research (India); Institutional Review Board, Institute of Medicine, Tribhuvan University; Ethical Review Board, Nepal Health Research Council; Institutional Review Board, Walter Reed Army Institute of Research (Nepal); Institutional Review Board, Johns Hopkins University; PRISMA Ethics Committee; Health Ministry, Loreto (Peru); Ethical Review Committee, Aga Khan University (PKN); Health, Safety and Research Ethics Committee, University of Venda; Department of Health and Social Development, Limpopo Provincial Government (South Africa); Medical Research Coordinating Committee, National Institute for Medical Research; Chief Medical Officer, Ministry of Health and Social Welfare (Tanzania). Informed written consent was obtained from the parent or guardian of each participating child on their behalf.

### Analysis of stool specimens

Total nucleic acid was extracted from stool specimens from children who completed 2 years of follow-up using the QIAmp Fast DNA Stool Mini Kit (Qiagen), as previously described [16]. Extrinsic controls phocine herpesvirus and bacteriophage MS2 monitored the efficiency of extraction and amplification. Quantitative PCR with custom-designed TaqMan Array Cards was used to detect 29 enteropathogens using the AgPath One Step realtime PCR kit (ThermoFisher), as described elsewhere [5,17]. *Shigella* was detected using the *ipaH* gene, and as in previous work [5,6], we interpret *ipaH* detections as diagnostic of *Shigella* even though both *Shigella* and enteroinvasive *E*. *coli* are detected using the *ipaH* target. *Shigella* species were detected using periplasmic protein, O-antigen, and type 3 restriction enzyme genes (*S*. *flexneri*) and a methylase gene (*S*. *sonnei*), as previously described [4]. *Shigella* infection was defined by *ipaH* qPCR cycle threshold (Cq) < 35, and quantity defined by log_10_-copy numbers per gram of stool based on the Cq, as previously [6]. Among *ipaH* positive stools, speciation assays were considered positive when Cq < 40 since the speciation assays target single copy genes and are therefore less sensitive than the *ipaH* assay. *Shigella*-attributable diarrhea episodes were identified using attributable fractions (AFe) to adjust for subclinical pathogen infections, as previously [4,5]. We defined *Shigella*-attributable episodes when the *Shigella* quantity-derived AFe ≥ 0.5 (i.e. majority attribution). *Shigella* was also previously detected by culture using standard protocols across sites in all diarrheal stools and non-diarrheal stools collected monthly in the first year and quarterly in the second year [18].

Fecal intestinal biomarkers were analyzed in the same subset of non-diarrheal stools, as described previously [19,20]. Myeloperoxidase (MPO; log [ng/ml]) was measured as marker of neutrophil activation and infiltration, neopterin (NEO; log[nmol/L]) as a marker of Th1 inflammation, and α-1-anti-trypsin (AAT; log[mg/g]) as a marker of intestinal permeability. Urinary lactulose and mannitol were measured at 3, 6, 9, and 15 months, and were converted into sample-based lactulose:mannitol excretion ratio z-scores (LMZs) using the Brazil cohort as the internal reference population [21]. α-1-acid glycoprotein (AGP), a marker of systemic inflammation, was measured in serum from 7, 15, and 24 months.

### Statistical analysis

We analyzed all stool samples with valid qPCR results for *Shigella* (97.1%, n = 41,450 of 42,630 samples with sufficient stool collected). We estimated the incidence of *Shigella*-attributable diarrhea (AFe ≥ 0.5) using Poisson regression and reweighted estimates from the number of episodes tested to the total number of episodes identified by surveillance. We estimated the relative risks of an episode presenting with each clinical characteristic in the first versus second year of life, and for children’s first versus subsequent *Shigella* diarrhea episodes, using log-binomial regression with generalized estimating equations (GEE) to account for correlated episodes within children, adjusting for site.

We assessed diagnostic test characteristics of *Shigella* culture compared to qPCR as the gold standard among all stools, among attributable diarrheal stools, and among attributable dysenteric stools by site. We further estimated the associations between clinical characteristics of attributable episodes and culture positivity using log-binomial regression with GEE and in univariable (adjusting for site) and multivariable (adjusting for site and other clinical characteristics) models.

To identify risk factors for *Shigella* infection in both non-diarrheal and diarrheal stools, we included sociodemographics, household, and child-level variables as potential risk factors in univariable log-binomial regression models with GEE, adjusting only for site. We then estimated adjusted associations in a multivariable model with a subset of risk factors that were either statistically significant (p<0.05) or had a risk ratio with magnitude greater than 1.2 or less than 0.83 in the univariable models, overall and by site. For covariates measuring similar constructs (e.g. anthropometric measurements and recent antibiotic use), we included the variables with complete data and/or larger magnitudes of effect in the multivariable model.

We modeled the seasonality of *Shigella* detections in non-diarrheal stools at each site using predictions from logistic regression models with linear and quadratic terms for the week of the year (*w*), and the terms sin(2π*w*/52), cos(2π*w*/52), sin(4π*w*/52), and cos(4π*w*/52). We estimated the associations of historical monthly average temperature and rainfall from 1982–2012 for towns nearest each site [22] with *Shigella* using log-binomial regression with effects scaled to compare high versus low temperature and rainfall at each site, defined by the 90^th^ and 10^th^ percentiles of the site-specific distributions.

Finally, we assessed the associations between concurrent measurements of biomarkers and *Shigella* infection and quantity, both in quartiles and continuously per log_10_ increase in copy numbers per gram of stool based on the Cq value, using linear regression models with GEE and adjusting for site, age, sex, and stool consistency.

## Results

### Shigella burden

Among 1715 children with 2 years of follow up, a total of 41,405 stool samples (6,751 diarrheal and 34,654 non-diarrheal samples) were tested for *Shigella* by qPCR (Table 1). The prevalence of *Shigella* was 11.5% (n = 4744) overall, and was higher in diarrheal stools than non-diarrheal stools (18.4% vs. 10.1%; p<0.0001). *Shigella* prevalence in non-diarrheal stools varied from 4.9%-17.8% across sites. Almost half (611/1407, 43.4%) of children with *Shigella* detected in one or more non-diarrheal stools never had a diarrhea episode in which *Shigella* was detected, and almost two-thirds (900/1407, 64.0%) never experienced a *Shigella-*attributable diarrhea episode. The quantity of *Shigella* detected was approximately 1 log higher in diarrheal (6.13 log_10_-copy numbers per gram of stool) compared to non-diarrheal (5.56 log_10_-copy numbers) stools, and quantities were lower in Pakistan and South Africa compared to the other sites. The prevalence and quantity of *Shigella* infection increased with age (Table 1).

**Table 1:** Prevalence of *Shigella* infection, quantity detected, and incidence of *Shigella*-attributable diarrhea among 1,715 children in the MAL-ED study.

	Prevalence in diarrheal stools (N = 6,751) n (%)	Quantity^1^ in diarrheal stools Mean (SD)	Prevalence in non-diarrheal stools (N = 34,654) n (%)	Quantity^1^ in non-diarrheal stools Mean (SD)	Number of *Shigella*-attributable diarrhea episodes	Incidence of *Shigella*-attributable diarrhea^1^ (95% CI)	Number of *Shigella*-attributable dysentery episodes	Incidence of *Shigella*-attributable dysentery^2^ (95% CI)
Overall	1239 (18.35)	6.13 (1.54)	3505 (10.11)	5.56 (1.28)	755	31.8 (29.6, 34.2)	111	4.85 (4.02, 5.84)
Site								
Dhaka, Bangladesh	402 (29.03)	6.19 (1.46)	564 (13.06)	5.61 (1.19)	275	75.1 (66.7, 84.5)	30	8.23 (5.75, 11.77)
Fortaleza, Brazil	21 (23.08)	6.18 (1.73)	139 (4.90)	5.14 (1.24)	12	6.7 (3.8, 11.8)	0	0
Vellore, India	141 (22.07)	6.43 (1.46)	592 (12.42)	5.47 (1.20)	101	33.9 (27.9, 41.1)	23	8.32 (5.53, 12.52)
Bhaktapur, Nepal	118 (12.98)	6.69 (1.81)	290 (5.75)	5.64 (1.26)	80	21.0 (16.9, 26.2)	16	3.93 (2.41, 6.41)
Loreto, Peru	305 (19.01)	6.28 (1.66)	574 (13.63)	5.81 (1.43)	162	47.9 (41, 55.8)	25	7.47 (5.04, 11.04)
Naushero Feroze, Pakistan	195 (10.57)	5.29 (1.00)	268 (5.77)	4.89 (0.89)	102	39.0 (32.1, 47.4)	12	4.51 (2.56, 7.93)
Venda, South Africa	20 (16.81)	5.47 (1.22)	321 (7.01)	5.05 (0.94)	10	5.4 (2.9, 10.0)	0	0
Haydom, Tanzania	37 (23.13)	6.12 (1.47)	757 (17.80)	5.91 (1.37)	13	10.8 (6.2, 18.5)	5	3.81 (1.58, 9.14)
Age (months)								
0–5	44 (2.67)	5.31 (1.34)	144 (1.87)	4.97 (1.08)	16	2.9 (1.8, 4.8)	4	0.78 (0.29, 2.09)
6–11	253 (12.22)	5.79 (1.55)	620 (7.39)	5.50 (1.23)	128	21.8 (18.3, 25.9)	19	3.31 (2.11, 5.18)
12–17	426 (25.10)	6.17 (1.55)	1096 (12.26)	5.52 (1.27)	265	44.8 (39.7, 50.5)	39	7.00 (5.11, 9.58)
18–23	516 (38.62)	6.33 (1.50)	1645 (17.08)	5.67 (1.31)	346	53.3 (48.0, 59.2)	49	7.66 (5.79, 10.14)

^1^log_10_-copy numbers per gram of stool based on the Cq value

^2^Episodes with AFe ≥ 0.5 per 100 child years, reweighted from episodes tested to total number of episodes surveilled

**Fig 1:**
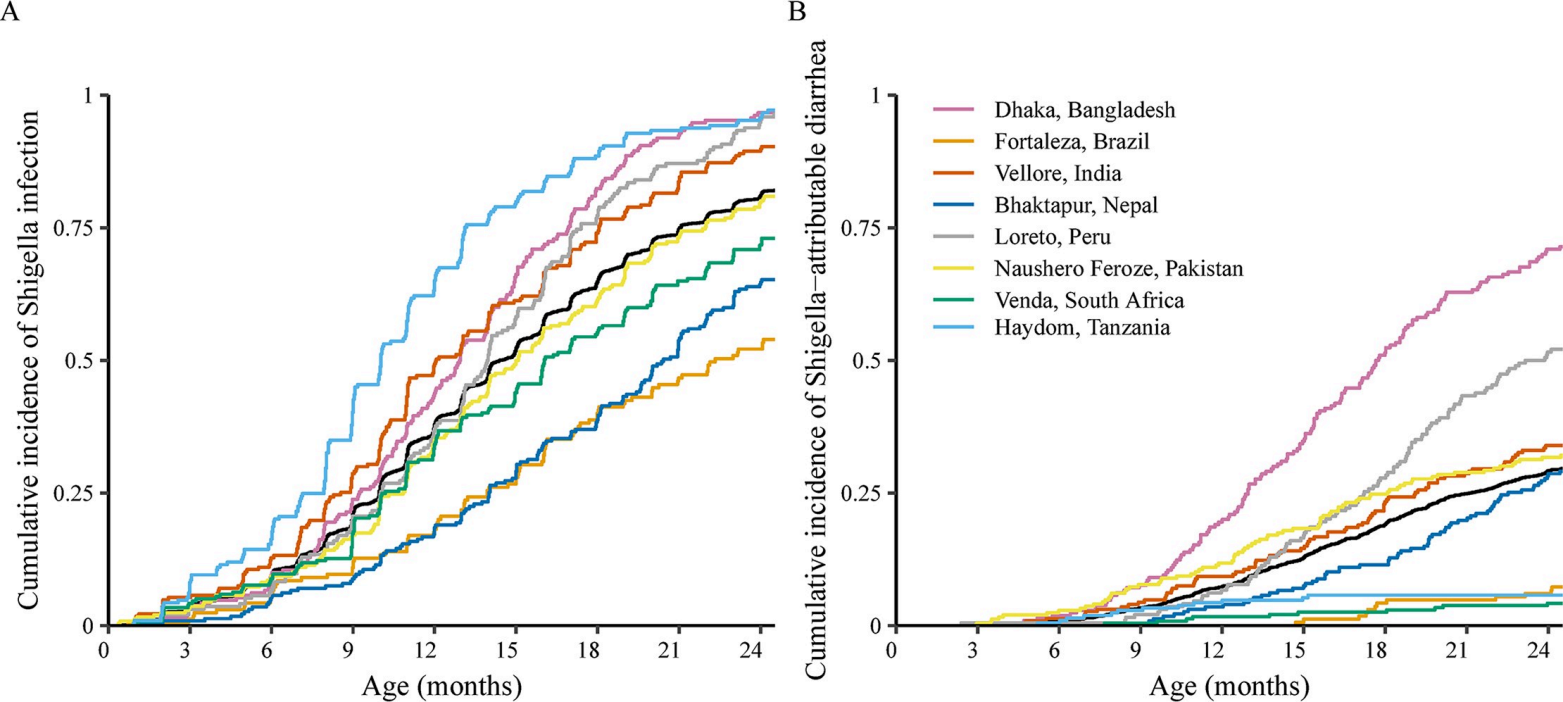
Cumulative incidence of *Shigella* infection (A) and *Shigella*-attributable diarrhea episodes (B) among 1715 children in the MAL-ED cohort.

*Shigella* was detected at quantities high enough to attribute the episode to *Shigella* in 11.2% (n = 755) of diarrheal stools. The overall incidence of *Shigella*-attributable diarrhea was 31.8 cases (95% CI: 29.6, 34.2) per 100 child-years. Tanzania had the third lowest incidence (10.8 cases per 100 child-years, 95% CI: 6.2, 18.5) of *Shigella*-attributable diarrhea despite having the highest prevalence in stools overall. Incidence of *Shigella*-attributable diarrhea was higher in older age groups (incidence rate ratio: 1.75, 95% CI: 1.70, 1.81 per 6-month increase in age). Incidence of *Shigella-*attributable dysentery was lower (4.85 cases per 100 child-years, 95% CI: 4.02, 5.84), but followed similar patterns by age and site. Incidence estimates by disease severity and diagnostic are reported in S1 Table. By two years of age, 82.0% (n = 1407) of children had been infected with *Shigella* (Fig 1A), and 29.6% (n = 507) had at least one episode of *Shigella-*attributable diarrhea (Fig 1B). Median time to first infection was 14.4 (95% CI: 14.0, 15.0) months.

Because the *Shigella* speciation assays were less sensitive than that for *ipaH*, results were available for only 31.3% (n = 1245) of *ipaH* positive stools with speciation testing (n = 3980, 83.9% of *ipaH* positives). *ipaH* quantity was 6.4 log_10_-copy numbers per gram of stool in speciated detections compared to 5.4 log_10_-copy numbers in non-speciated detections. Among the speciated *Shigella*-attributable diarrheal stools (n = 258), 58.9% (n = 152) were *S*. *flexneri* and 43.8% (n = 113) were *S*. *sonnei*. The majority of *Shigella*-attributable diarrheal stools were *S*. *flexneri* in all sites except Brazil, Nepal, and South Africa. The ratio of *S*. *flexneri* to *S*. *sonnei* (71.9% vs. 31.7%) observed in all (diarrheal and non-diarrheal) stools was similar to that in diarrheal stools (S2 Table).

### Clinical characteristics of Shigella diarrhea

Among *Shigella*-attributable diarrheal episodes, 28.3% (n = 214) were severe and 14.7% (n = 111) had bloody stools. Approximately a third (n = 235, 31.1%) were accompanied by fever, 18.5% (n = 140) by vomiting, 10.1% (n = 76) by dehydration, and 18.9% (n = 143) lasted 7 days or longer. The majority of episodes were antibiotic treated (456, 60.4%), and of these, the majority were treated with a macrolide, cephalosporin, or fluoroquinolone (n = 311/456, 68.2%). However, there was substantial site variability; 97.0% of all macrolide treatment was given in Bangladesh and Peru, and antibiotic treatment was rare in Brazil and South Africa. There were differences in the clinical presentation of shigellosis by age (Table 2). Episodes of *Shigella*-attributable diarrhea in the first year of life were more likely to be prolonged (aRR: 1.24, 95% CI: 0.88, 1.74), with vomiting (aRR: 1.72, 95% CI: 1.26, 2.35) and with more than 6 loose stools in 24 hours (aRR: 1.41, 95% CI: 1.06, 1.86) compared to episodes in the second year. Episodes in the first year were also more likely to be treated with cephalosporins and less likely to be treated with fluoroquinolones. Adjusting for age, a child’s first episode of *Shigella*-attributable diarrhea was more likely to be accompanied by dehydration (aRR: 1.41, 95% CI: 0.84, 2.36) compared to subsequent episodes. First episodes were also slightly more likely to be prolonged and with vomiting and high frequency of stools, but less likely to be bloody (S3 Table).

**Table 2:** Clinical characteristics of *Shigella*-attributable diarrhea episodes in the first and second years of life and the associations between age and clinical characteristics among 755 episodes.

Episode characteristic	Year 1 (0–11 months; N = 144) N (%)	Year 2 (12–23 months; N = 611) N (%)	Risk ratio^1^ for characteristic in year 1 vs. year 2 (95% CI)
Severe (score ≥4)	47 (32.6)	167 (27.3)	1.16 (0.89, 1.50)
Blood	23 (16.0)	88 (14.4)	1.06 (0.69, 1.63)
Fever	48 (33.3)	187 (30.6)	1.04 (0.81, 1.33)
Prolonged (≥7 days)	35 (24.3)	108 (17.7)	1.24 (0.88, 1.74)
Persistent (≥14 days)	8 (5.6)	18 (2.9)	1.32 (0.59, 2.93)
Dehydration	18 (12.5)	58 (9.5)	1.11 (0.72, 1.72)
Vomiting	44 (30.6)	96 (15.7)	1.72 (1.26, 2.35)
High frequency (>6 loose stools in 24 hours)	43 (29.9)	137 (22.4)	1.41 (1.06, 1.86)
Hospitalization	1 (0.7)	1 (0.2)	—
Any antibiotic treatment^2^	88 (61.1)	368 (60.2)	0.97 (0.83, 1.12)
Macrolide treatment^2^	29 (20.1)	136 (22.3)	1.05 (0.76, 1.45)
Cephalosporin treatment^2^	15 (10.4)	44 (7.2)	1.21 (0.72, 2.02)
Fluoroquinolone treatment^2^	10 (6.9)	97 (15.9)	0.46 (0.25, 0.85)

^1^Adjusted for site; excludes sites with no *Shigella*-attributable diarrhea episodes with characteristic (Brazil for severe, dehydration, and high frequency; Brazil and South Africa for blood; Brazil, South Africa, and Tanzania for persistent diarrhea, macrolide, cephalosporin, and fluoroquinolone treatment).

^2^Treatment on at least one day during the diarrhea episode.

Coinfections were detected in almost all *Shigella*-attributable diarrheal stools (S4 Table). However, a second etiology of diarrhea (i.e. a coinfecting pathogen was detected in a quantity high enough to be associated with diarrhea) was identified in only 38.5% of these episodes (n = 289). The coinfecting pathogen had a higher AFe than *Shigella* (i.e. potentially the primary etiology) in 12.3% of episodes (n = 92). Episodes with a viral co-etiology were less likely to be bloody (aRR: 0.60, 95% CI: 0.38, 0.96) and more likely to include vomiting (aRR: 1.79, 95% CI: 1.31, 2.46) compared to episodes with *Shigella* as the only etiology identified. Episodes with another bacterial co-etiology were also less likely to be bloody (aRR: 0.62, 95% CI: 0.35, 1.11; S5 Table).

### Performance of Shigella culture

Of 30,678 stools tested by both qPCR and culture, 3,372 (11.0%) were positive by qPCR and 280 (0.9%) were positive by culture. Considering qPCR the gold standard, the overall sensitivity of culture was 6.6% (Table 3). Specificity was uniformly high, at more than 99% at all sites. In the subset of *Shigella*-attributable diarrheal stools (n = 736), which have higher *Shigella* quantity detected than all stools, sensitivity improved to 17.5%, and ranged from 0% in South Africa and Tanzania to 24.0% in Peru (Table 3). Sensitivity was even higher in dysentery episodes (27.8%). The sensitivity of culture among *Shigella*-attributable diarrheal stools without another attributable pathogen identified (20.6%, n = 94/457) was higher than that among *Shigella*-attributable diarrheal stools with another attributable pathogen identified (12.5%, n = 35/279). Culture positivity was strongly associated with age, such that attributable diarrhea stools among younger children were less likely to be culture positive (Table 4). Presence of blood had the strongest association with culture positivity (aRR: 1.84, 95% CI: 1.28, 2.65), but culture still missed more than 70% of dysentery cases. Diarrhea severity was not associated with culture positivity, and caregiver-reported fever was inversely associated with culture positivity. Recent macrolide treatment was also associated with reduced detection by culture (aRR: 0.57, 95% CI: 0.30, 1.08; Table 4).

**Table 3:** Performance of *Shigella* culture compared to quantitative PCR among 30,678 diarrheal and non-diarrheal stools tested by both diagnostics.

	All sites n (%)	Dhaka, Bangladesh n (%)	Fortaleza, Brazil n (%)	Vellore, India n (%)	Bhaktapur, Nepal n (%)	Loreto, Peru n (%)	Naushero Feroze, Pakistan n (%)	Venda, South Africa n (%)	Haydom, Tanzania n (%)
All stools	30678	4305	1895	3722	4176	5809	4716	3158	2897
qPCR positive	3372 (11.0)	667 (15.5)	95 (5.0)	448 (12.0)	258 (6.2)	879 (15.1)	323 (6.8)	204 (6.5)	498 (17.2)
Culture positive	280 (0.9)	34 (0.8)	5 (0.3)	45 (1.2)	53 (1.3)	56 (1)	79 (1.7)	3 (0.1)	5 (0.2)
Sensitivity^1^	6.6	3.6	4.2	8.7	16.7	6.3	17.3	0.5	0.2
Specificity^1^	99.8	99.7	99.9	99.8	99.7	99.9	99.5	99.9	99.8
Positive predictive value^1^	79.6	70.6	80.0	86.7	81.1	98.2	70.9	33.3	20.0
Negative predictive value^1^	89.6	84.9	95.2	88.9	94.8	85.7	94.2	93.6	82.8
*Shigella*-attributable diarrhea episodes	736	267	11	101	76	162	97	10	12
Culture positive^2^	129 (17.5)	14 (10.9)	3 (2.3)	24 (18.6)	29 (22.5)	31 (24.0)	28 (21.7)	0	0
*Shigella*-attributable dysentery episodes	108	29	0	23	15	25	11	0	5
Culture positive^2^	30 (27.8)	3 (10.3)	0	6 (26.1)	6 (40.0)	11 (44.0)	4 (36.4)	0	0

^1^qPCR considered the gold standard

^2^Number of *Shigella-*attributable episodes (defined by qPCR positive with AFe ≥ 0.5) that were also culture positive; percent positive is the sensitivity of culture compared to qPCR as the gold standard

**Table 4:** Associations between characteristics of *Shigella*-attributable diarrhea episodes and culture positivity among 736 attributable diarrhea episodes tested by qPCR and culture^1^.

Episode characteristic	N episodes	N (%) culture positive	Univariable^2^ Risk ratio (95% CI)	Multivariable^3^ Risk ratio (95% CI)
Age (months)				
0–5	15	1 (6.7)	0.25 (0.04, 1.70)	0.19 (0.02, 1.51)
6–11	116	13 (11.2)	0.53 (0.31, 0.92)	0.51 (0.30, 0.88)
12–17	246	44 (17.9)	0.82 (0.59, 1.14)	0.86 (0.62, 1.20)
18–23	326	68 (20.9)	1.	1.
Severe (score ≥4)	211	35 (16.6)	0.91 (0.64, 1.28)	--^3^
Blood	103	30 (29.1)	1.53 (1.09, 2.15)	1.84 (1.28, 2.65)
Fever	225	35 (15.6)	0.69 (0.47, 1.01)	0.66 (0.45, 0.95)
Prolonged (≥7 days)	137	29 (21.2)	0.88 (0.60, 1.29)	0.84 (0.55, 1.29)
Persistent (≥14 days)	26	6 (23.1)	0.89 (0.40, 1.96)	--^3^
Dehydration	71	20 (28.2)	1.22 (0.80, 1.86)	1.17 (0.77, 1.78)
Vomiting	131	22 (16.8)	0.89 (0.59, 1.36)	1.13 (0.73, 1.75)
No. loose stools (mean; SD)	5.8 (2.64)	6.0 (2.37)	1.01 (0.96, 1.06)	1.03 (0.97, 1.09)
Any antibiotic treatment in the last 15 days	420	65 (15.5)	0.87 (0.63, 1.20)	--^3^
Macrolide treatment in the last 15 days	146	10 (6.9)	0.64 (0.33, 1.22)	0.57 (0.30, 1.08)
Cephalosporin treatment in the last 15 days	66	14 (21.2)	1.04 (0.64, 1.69)	0.95 (0.57, 1.60)
Fluoroquinolone treatment in the last 15 days	77	9 (11.7)	0.95 (0.52, 1.73)	0.71 (0.38, 1.32)

^1^Analysis excludes Brazil, South Africa, and Tanzania, which had 5 or fewer culture-positive attributable diarrhea episodes

^2^Adjusted for site

^3^Adjusted for other characteristics included in the table; estimates are excluded for composite (collinear) variables

### Risk factors for Shigella

In univariable analysis, unimproved sanitation, crowding (2+ people living in a single room), <10 years of maternal education, having 3+ live children, initiating complementary foods before 3 months, recent diarrhea, antibiotic use (particularly fluoroquinolone use), and lower anthropometric measurements prior to sample collection were also associated with higher risk of *Shigella* infection (Table 5). Of these variables, unimproved sanitation (aRR: 1.15, 95% CI: 1.03, 1.29), less than 10 years of maternal education (aRR: 1.14, 95% CI: 1.03, 1.26), and child WAZ at the most recent measurement prior to diarrhea (aRR: 0.91, 95% CI: 0.88, 0.95 per z-score increase in weight) were the strongest risk factors in multivariable analysis (Table 5). There was some variability in associations by site; for example, crowding and recent diarrhea were strong risk factors in Brazil, low maternal education had the largest associations in Brazil, India, and South Africa, and recent fluoroquinolone use was only a risk factor in India and Pakistan (S1 Fig).

**Table 5:** Risk factors for *Shigella* infection among 41,405 diarrheal and non-diarrheal stools.

	Univariate^1^ analysis			Multivariate^2^ analysis		
	Risk ratio (95% CI)		P-value	Risk ratio (95% CI)		P-value
Diarrheal stool (Ref: non-diarrheal stool)	1.86	(1.74, 1.99)	<0.0001	1.97	(1.85, 2.10)	<0.0001
**Household level factors**						
Exchange rate adjusted income < $150 dollar (Ref: income > = $150)	1.07	(0.97, 1.17)	0.2			
Earth, sand, clay, mud, dung floor (Ref: Wooden, concrete, vinyl etc.)	1.09	(0.98, 1.23)	0.1			
Unimproved sanitation	1.21	(1.09, 1.35)	0.0005	1.15	(1.03, 1.29)	0.01
Unimproved source of drinking water	1.05	(0.93, 1.18)	0.4			
Crowding (2+ people per room)	1.09	(1.00, 1.19)	0.05	1.04	(0.95, 1.14)	0.4
Ownership of cattle	1.01	(0.90, 1.13)	0.9			
Ownership of chicken	0.98	(0.88, 1.09)	0.7			
**Maternal characteristics**						
<10 years of maternal education	1.19	(1.08, 1.32)	0.0005	1.14	(1.03, 1.26)	0.01
Maternal BMI (Ref. 18.5–22.9 kg/m^2^)						
<18.5 kg/m^2^	1.10	(0.96, 1.25)	0.2			
≥23 kg/m^2^	1.02	(0.95, 1.11)	0.6			
3 or more living children	1.08	(1.00, 1.17)	0.05	1.01	(0.94, 1.10)	0.7
**Child characteristics**						
Age (months) (Ref. 0–5 months)						
6–11	4.13	(3.49, 4.88)	<0.0001	3.93	(3.32, 4.64)	<0.0001
12–17	7.04	(5.95, 8.32)	<0.0001	6.84	(5.78, 8.09)	<0.0001
18–23	9.78	(8.30, 11.53)	<0.0001	9.66	(8.19, 11.39)	<0.0001
Female sex (Ref: Male)	1.00	(0.93, 1.07)	1.0			
Exclusive breastfeeding < 3 months	1.09	(1.00, 1.19)	0.06			
Complementary foods initiated < 3 months	1.11	(1.02, 1.21)	0.02	1.10	(1.01, 1.20)	0.03
WAZ at enrollment	0.99	(0.96, 1.03)	0.7			
LAZ at enrollment^3^	0.99	(0.96, 1.03)	0.7			
WLZ^3^	0.80	(0.77, 0.83)	<0.0001			
WAZ	0.79	(0.76, 0.82)	<0.0001	0.91	(0.88, 0.95)	<0.0001
LAZ^3^	0.73	(0.71, 0.76)	<0.0001			
Days with diarrhea (past 3 months; per 7 day increase)	1.09	(1.05, 1.13)	<0.0001	1.06	(1.02, 1.09)	0.002
Antibiotic use (past 15 days)	1.13	(1.06, 1.20)	0.0002			
Antibiotic use (past 30 days)	1.02	(0.96, 1.09)	0.6			
Macrolide use (past 15 days)	1.01	(0.89, 1.14)	0.9			
Macrolide use (past 30 days)	0.92	(0.81, 1.05)	0.2			
Cephalosporin use (past 15 days)	0.97	(0.85, 1.11)	0.7			
Cephalosporin use (past 30 days)	0.89	(0.77, 1.01)	0.08			
Fluoroquinolone use (past 15 days)^4^	1.67	(1.43, 1.94)	<0.0001			
Fluoroquinolone use (past 30 days)	1.54	(1.29, 1.86)	<0.0001	1.10	(0.93, 1.31)	0.3

^1^Adjusted for site.

^2^Adjusted for site and other variables in table with multivariate estimates.

^3^Excludes Pakistan.

^4^Not included in multivariable model since collinear with fluoroquinolone use in past 30 days.

**Fig 2:**
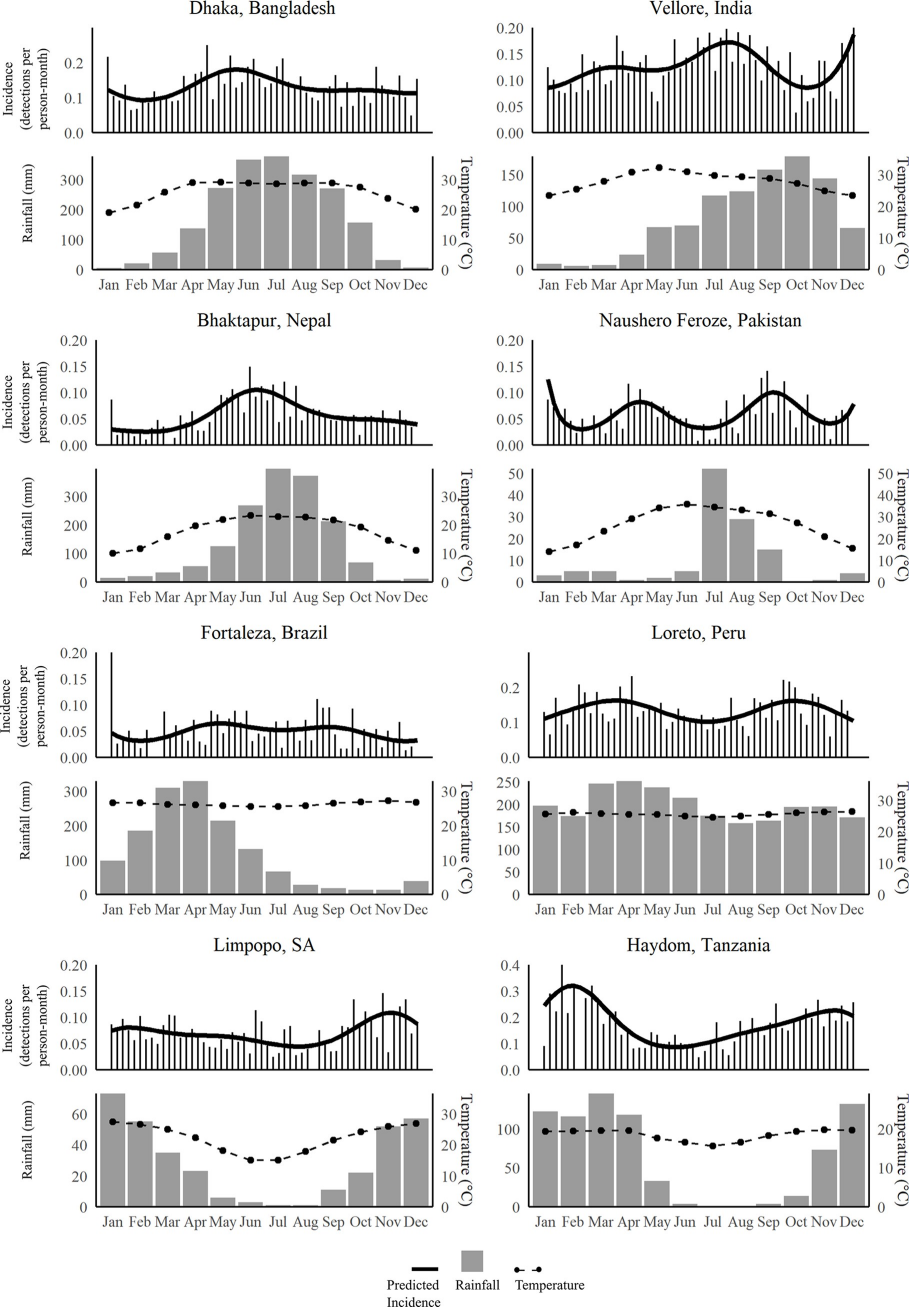
Seasonality of *Shigella* detections in non-diarrheal stools by site. Top plots: weekly incidence (bars) and modeled incidence (solid dark lines); bottom plots: historical monthly averages for rainfall (bars) and temperature (dotted lines) at each sites.

The seasonality of *Shigella* infections differed by site (Fig 2). Peak prevalence was observed in May/June in Bangladesh, June in Nepal, July/August in India, November in South Africa, and February in Tanzania. Two peaks were observed in Pakistan and Peru, and there was little seasonality in Brazil. Among climactic factors that could explain these heterogeneities, temperature was more strongly associated with *Shigella* detection than rainfall, though temperature was collinear with rainfall at most sites (Fig 2, S6 Table). Higher temperature was most strongly associated with *Shigella* in Nepal (aRR: 2.71, 95% CI: 1.96, 3.75) and Tanzania (aRR: 1.92, 95% CI: 1.63, 2.27). Uniquely, higher rainfall was protective in Pakistan (aRR: 0.78, 95% CI: 0.61, 1.00; S6 Table).

### Association of Shigella with biomarkers of environmental enteropathy

Myeloperoxidase levels were 0.33 log(ng/mL) (95% CI: 0.27, 0.40) higher in stools with *Shigella*, and the association was greater in diarrheal stools (mean difference: 0.65, 95% CI: 0.30, 1.00 log(ng/ml)) than in non-diarrheal stools (mean difference: 0.32, 95% CI: 0.26, 0.39; p for heterogeneity: 0.08). There was a linear dose-response with *Shigella* quantity; myeloperoxidase levels increased by 0.21 logs (0.16, 0.26) per log increase in *Shigella* quantity (Table 6). Associations with myeloperoxidase were observed in all sites, but were highest in Brazil (0.70, 95% CI: 0.29, 1.11) and Peru (0.56, 95% CI: 0.40, 0.71; S7 Table). The dose response relationship was also consistent across sites (S2 Fig). α-1-acid glycoprotein was slightly elevated in stools with *Shigella* (7.53 mg/dL difference, 95% CI: 3.51, 11.55), and higher *Shigella* quantity was associated with higher concentrations. There were no consistent associations with neopterin, α-1-antitrypsin, the lactulose:mannitol z-score, or its components (Table 6).

**Table 6:** Associations between *Shigella* and biomarkers of environmental enteropathy among 19,148 diarrheal and non-diarrheal stools with biomarker measurements.

	Adjusted^1^ concentration difference (95% CI)						
	Myeloperoxidase (log[ng/mL])	Neopterin (log[nmol/L])	α-1-antitrypsin (log[mg/g])	α-1-acid glycoprotein (mg/dL)^2^	Lactulose:mannitol z-score^3^	Lactulose z-score^3^	Mannitol z-score^3^
Any *Shigella*	0.33 (0.27, 0.40)	0.00 (-0.06, 0.06)	-0.01 (-0.07, 0.05)	7.53 (3.51, 11.55)	0.09 (-0.02, 0.21)	-0.05 (-0.19, 0.08)	-0.12 (-0.24, -0.01)
*Shigella* quantity							
1^st^ quartile	0.09 (-0.02, 0.21)	-0.02 (-0.14, 0.10)	-0.03 (-0.14, 0.07)	1.58 (-4.07, 7.23)	0.17 (0.01, 0.34)	0.03 (-0.19, 0.25)	-0.14 (-0.33, 0.06)
2^nd^ quartile	0.19 (0.07, 0.30)	0.01 (-0.10, 0.12)	-0.05 (-0.16, 0.05)	7.60 (0.82, 14.38)	0.07 (-0.16, 0.30)	-0.19 (-0.43, 0.05)	-0.20 (-0.40, 0.01)
3^rd^ quartile	0.43 (0.31, 0.54)	-0.03 (-0.16, 0.09)	-0.02 (-0.12, 0.09)	9.28 (1.70, 16.86)	-0.11 (-0.37, 0.14)	-0.07 (-0.33, 0.19)	0.05 (-0.17, 0.26)
4^th^ quartile	0.81 (0.66, 0.95)	0.07 (-0.05, 0.20)	0.10 (-0.01, 0.22)	12.90 (4.81, 21.00)	0.26 (0.06, 0.45)	0.01 (-0.25, 0.28)	-0.23 (-0.45, -0.02)
Per log increase	0.21 (0.16, 0.26)	0.06 (0.01, 0.11)	0.03 (-0.01, 0.07)	2.19 (-0.35, 4.74)	0.04 (-0.03, 0.12)	0.07 (-0.02, 0.16)	-0.01 (-0.08, 0.06)

^1^Adjusted for site, age, sex, and stool consistency.

^2^N = 4147 at 7, 15, and 24 months of age; estimates adjusted for site, age, and sex.

^3^Brazil cohort was the internal reference population; N = 6110 at 3, 6, 9, and 15 months of age; estimates adjusted for site, age, and sex.

## Discussion

The burden of *Shigella* among children under two was heterogeneous across eight sites with the absolute burden of infection and illness was higher in the South Asian sites and Peru. The burden of *Shigella* diarrhea relative to subclinical infections also varied. *Shigella* diarrhea episodes were accompanied by blood in only a minority of episodes, and episodes were generally more severe in the younger children. In a minority of *Shigella* diarrhea episodes that were also attributable to another pathogen, clinical phenotypes were often mixed; for example, episodes with viral co-etiologies predictably presented with more vomiting.

An 11-fold higher detection of *Shigella* was observed with qPCR compared to culture, including a 3-fold increase for *Shigella*-attributable dysentery. While higher sensitivity of culture among more severe cases has been previously noted [23], culture still missed the majority of cases of *Shigella* diarrhea, severe diarrhea, and dysentery, and culture had the lowest sensitivity among young children who are at highest risk for poor outcomes. These results highlight the need for more sensitive diagnostic tools.

The analyses of *Shigella* risk factors were consistent with prior work, which identified maternal education, exclusive breastfeeding, and larger WAZ as protective [24–26], and found similar trends with age [24,26,27]. Undernourished children were more likely to be infected, and interestingly, the seasonality of *Shigella* in Tanzania mirrored the seasonality of malnutrition, previously described [28]. The identification of unimproved sanitation as a risk factor alongside seasonal patterns that correlate strongly with average temperature and rainfall suggest environmental transmission pathways may be important. The seasonal patterns also support the potential implication of houseflies as a mechanical vector, as housefly population densities are seasonal and have been shown to correlate with *Shigella* [29]. Surprisingly, recent antibiotic exposure, including to macrolides and fluoroquinolones which are recommended for the treatment of shigellosis [3], was not associated with reduced *Shigella* detection.

Among several biomarkers that indirectly measure environmental enteric dysfunction (EED), especially MPO, but also AGP, were elevated during *Shigella* infections with a dose-response with *Shigella* quantity. Several previous studies found that high levels of MPO were most predictive of linear growth decrements compared to other biomarkers, including in previous analyses of data from the Bangladesh [30] and Peru [31] MAL-ED sites, and in a birth cohort in Pakistan [32]. The associations between *Shigella* and MPO, and MPO and growth faltering, suggest a potential mechanism for the impact of *Shigella* on linear growth previously characterized [6].

This study was limited by the fact that stool samples were not collected and/or tested from all diarrhea episodes [5], such that we may have underestimated the incidence of *Shigella* diarrhea. Because a second etiology of diarrhea was frequently identified, we were unable to determine whether *Shigella* was the primary cause in a substantial subset of *Shigella-*attributable diarrhea episodes. Both *Shigella* and enteroinvasive *E*. *coli* can be detected using the *ipaH* gene. However, previous speciation [4] and metagenomic work [33] supports the interpretation of these detections as *Shigella*. In addition, the *Shigella* speciation assays were insensitive, such that species data were available for only a third of infections. Improvements to the speciation assays have been made since the MAL-ED study; validated real time PCR assays that can differentiate >80% *ipaH* positives regardless of culture positivity and identify a panel of *S*. *flexneri* serotypes (including 2a, 3a, and 6) are now available for future studies. Because deaths were rare in this community-based cohort, we could not assess the associations between *Shigella* and mortality, as in the GEMS study [34]. Finally, site-specific estimates were relatively imprecise given the low numbers of children at each site. Because burden varied substantially by site, the incidence estimates may not be generalizable to other low-resource settings.

The high burden of *Shigella* disease documented in MAL-ED highlights the potential utility of *Shigella* vaccines. Almost all children were exposed to *Shigella* by two years of age in most sites, which suggests a pathogen-specific population-based prevention strategy is warranted. Furthermore, because the incidence of *Shigella* diarrhea was higher in the second year of life, the vaccine could potentially be given later in infancy and prevent the majority of cases. However, younger children presented with more severe symptoms, suggesting protection early in infancy may be important. Continued monitoring of *Shigella* epidemiology is needed since incidence trends may change as macrolide antibiotics become more available globally. More than 15 *Shigella* vaccines are currently in development [10], with some rapidly advancing to evaluation in target populations. Because of the poor sensitivity of culture, use of molecular diagnostics to define outcomes in future vaccine efficacy studies could limit misclassification of the outcome and reduce the sample size required to estimate significant effects.

## Supporting information

S1 Checklist STROBE checklist. (PDF)

S1 Fig Site-specific associations between risk factors and *Shigella* detection in 41,405 diarrheal and non-diarrheal stools. Estimates are adjusted for age, diarrheal vs. non-diarrheal stool, and all other factors included in the figure. Estimates are excluded for specific sites for factors with no variability at that site.
(PDF)

S2 Fig Site-specific associations between *Shigella* quantity detected and myeloperoxidase (MPO) concentration among 19,146 diarrheal and non-diarrheal stools with MPO measurements. Estimates are adjusted for age, sex, and stool consistency.
(PDF)

S1 Table Incidence of *Shigella*-attributable diarrhea by disease severity and diagnostic among 1,715 children in the MAL-ED study. (PDF)

S2 Table Prevalence of *Shigella* species among 3,505 non-diarrheal, 1,239 diarrheal, and 755 attributable diarrheal stools with *Shigella* detected. (PDF)

S3 Table Clinical characteristics of *Shigella*-attributable diarrhea comparing children’s first episodes to subsequent episodes among 755 episodes. (PDF)

S4 Table Coinfections during 755 *Shigella*-attributable diarrhea episodes. (PDF)

S5 Table Clinical characteristics of *Shigella*-attributable diarrhea comparing episodes with *Shigella* as the only etiology identified to episodes with another etiology identified among 755 episodes. (PDF)

S6 Table Association between *Shigella* detection and historical monthly average temperature and rainfall from 1982–2012 by site. (PDF)

S7 Table Site-specific associations between *Shigella* and biomarkers of environmental enteropathy among 19,148 diarrheal and non-diarrheal stools with biomarker measurements. (PDF)
