# Supplementary material for: Epidemiology of Shigella infections and diarrhea in the first two years of life using culture-independent diagnostics in 8 low-resource settings
Source: PLoS Negl Trop Dis. 2020 Aug 17;14(8):e0008536. doi: 10.1371/journal.pntd.0008536 (PMC7451981; doi:10.1371/journal.pntd.0008536)
Supplement: S1 Fig — Estimates are adjusted for age, diarrheal vs. non-diarrheal stool, and all other factors included in the figure. Estimates are excluded for specific sites for factors with no variability at that site. (PDF) [file pntd.0008536.s002.pdf]

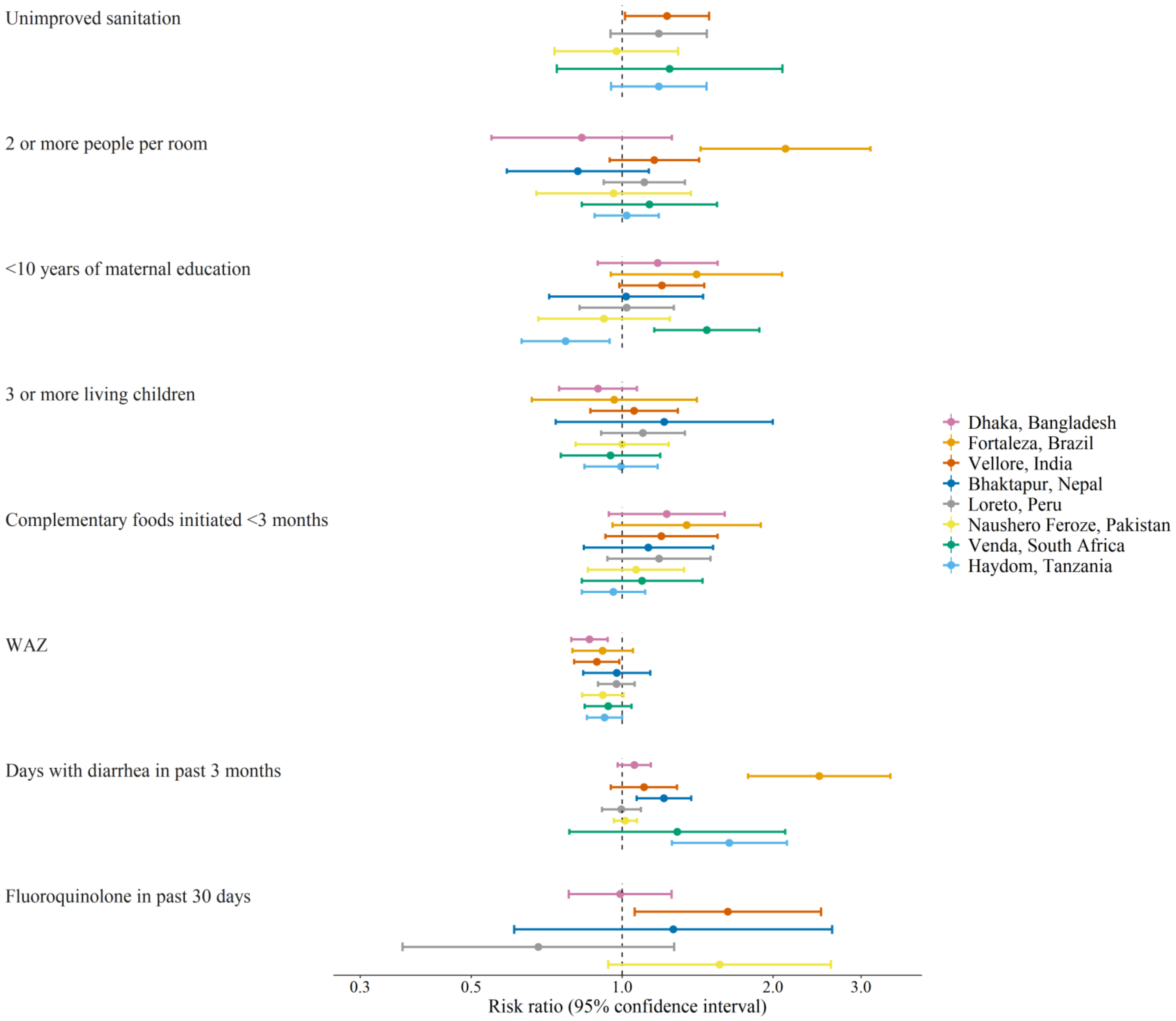

Figure S1. Site-specific associations between risk factors and *Shigella* detection in 41,405 diarrheal and non-diarrheal stools. Estimates are adjusted for age, diarrheal vs. non-diarrheal stool, and all other factors included in the figure. Estimates are excluded for specific sites for factors with no variability at that site.
