# Supplementary material for: Epidemiology of Shigella infections and diarrhea in the first two years of life using culture-independent diagnostics in 8 low-resource settings
Source: PLoS Negl Trop Dis. 2020 Aug 17;14(8):e0008536. doi: 10.1371/journal.pntd.0008536 (PMC7451981; doi:10.1371/journal.pntd.0008536)
Supplement: S2 Fig — Estimates are adjusted for age, sex, and stool consistency. (PDF) [file pntd.0008536.s003.pdf]

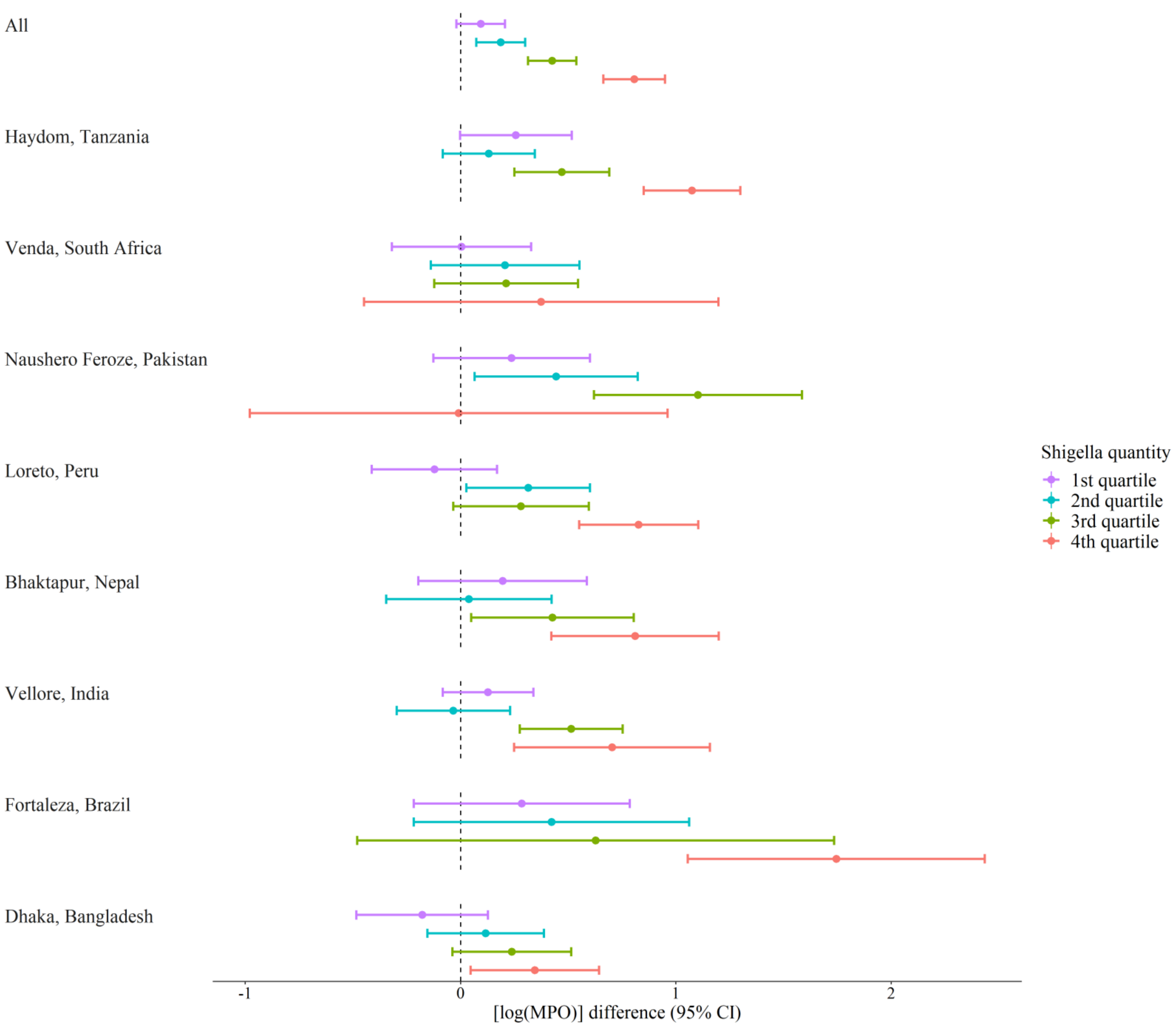

Figure S2. Site-specific associations between *Shigella* quantity detected and myeloperoxidase (MPO) concentration among 19,146 diarrheal and non-diarrheal stools with MPO measurements. Estimates are adjusted for age, sex, and stool consistency.
