# Supplementary material for: Epidemiology of Shigella infections and diarrhea in the first two years of life using culture-independent diagnostics in 8 low-resource settings
Source: PLoS Negl Trop Dis. 2020 Aug 17;14(8):e0008536. doi: 10.1371/journal.pntd.0008536 (PMC7451981; doi:10.1371/journal.pntd.0008536)
Supplement: S1 Table — (PDF) [file pntd.0008536.s004.pdf]

Table S1. Incidence of *Shigella*-attributable diarrhea by disease severity and diagnostic among 1,715 children in the MAL-ED study.

	Number of *Shigella*-attributable diarrhea episodes	Incidence of *Shigella*-attributable diarrhea[1] (95% CI)
Severe episodes[2]		
CODA $\geq$ 4	214	8.2 (7.1, 9.3)
Modified Vesikari[3] > 6	90	3.4 (2.7, 4.1)
Moderate-to-severe[4]	166	7.0 (6.0, 8.1)
Diagnostic		
qPCR (AFe $\geq$ 0.5)	755	31.8 (29.6, 34.2)
qPCR (any detection; Cq < 35)	1239	52.2 (49.4, 55.2)
Culture positive	168	6.7 (5.8, 7.8)

[1]Episodes per 100 child years, reweighted from episodes tested to total number of episodes surveilled
[2]Episodes identified by qPCR with AFe $\geq$ 0.5
[3]Score derived from observed components of the Vesikari score [1]
[4]Moderate-to-severe as defined in the Global Enteric Multicenter Study (GEMS) [2]

References

1. Platts-Mills JA, Babji S, Bodhidatta L, Gratz J, Haque R, Havt A, et al. Pathogen-specific burdens of community diarrhoea in developing countries: a multisite birth cohort study (MAL-ED). Lancet Glob Health. 2015;3: e564-75. doi:10.1016/S2214-109X(15)00151-5

2. Liu J, Platts-Mills JA, Juma J, Kabir F, Nkeze J, Okoi C, et al. Use of quantitative molecular diagnostic methods to identify causes of diarrhoea in children: a reanalysis of the GEMS case-control study. Lancet. 2016;388: 1291–1301. doi:10.1016/S0140-6736(16)31529-X
