# Supplementary material for: Epidemiology of Shigella infections and diarrhea in the first two years of life using culture-independent diagnostics in 8 low-resource settings
Source: PLoS Negl Trop Dis. 2020 Aug 17;14(8):e0008536. doi: 10.1371/journal.pntd.0008536 (PMC7451981; doi:10.1371/journal.pntd.0008536)
Supplement: S2 Table — (PDF) [file pntd.0008536.s005.pdf]

Table S2. Prevalence of *Shigella* species among 3,505 non-diarrheal, 1,239 diarrheal, and 755 attributable diarrheal stools with *Shigella* detected.

	Non-diarrheal stools				Diarrheal stools				*Shigella*-attributable diarrheal stools			
Site	N	Species data available[1] n (%)	*S. flexneri* n (%)	*S. sonnei* n (%)	N	Species data available[1] n (%)	*S. flexneri* n (%)	*S. sonnei* n (%)	N	Species data available[1] n (%)	*S. flexneri* n (%)	*S. sonnei* n (%)
Dhaka, Bangladesh	564	129 (22.9)	99 (76.7)	39 (30.2)	402	93 (23.1)	53 (57.0)	44 (47.3)	275	83 (30.2)	44 (53.0)	42 (50.6)
Fortaleza, Brazil	139	18 (12.9)	6 (33.3)	12 (66.7)	21	9 (42.9)	3 (33.3)	7 (77.8)	12	8 (66.7)	2 (25.0)	7 (87.5)
Vellore, India	592	162 (27.4)	114 (70.4)	50 (30.9)	141	21 (14.9)	13 (61.9)	10 (47.6)	101	21 (20.8)	13 (61.9)	10 (47.6)
Bhaktapur, Nepal	290	96 (33.1)	61 (63.5)	38 (39.6)	118	37 (31.4)	17 (45.9)	20 (54.1)	80	34 (42.5)	15 (44.1)	19 (55.9)
Loreto, Peru	574	140 (24.4)	105 (75.0)	39 (27.9)	305	92 (30.2)	69 (75.0)	24 (26.1)	162	69 (42.6)	51 (73.9)	19 (27.5)
Naushero Feroze, Pakistan	268	43 (16.0)	32 (74.4)	12 (27.9)	195	36 (18.5)	24 (66.7)	12 (33.3)	102	33 (32.4)	21 (63.6)	12 (36.4)
Venda, South Africa	321	56 (17.4)	37 (66.1)	20 (35.7)	20	4 (20.0)	1 (25.0)	3 (75.0)	10	4 (40.0)	1 (25.0)	3 (75.0)
Haydom, Tanzania	757	295 (39.0)	248 (84.1)	64 (21.7)	37	14 (37.8)	13 (92.9)	1 (7.1)	13	6 (46.2)	5 (83.3)	1 (16.7)
All	3505	939 (26.8)	702 (74.8)	274 (29.2)	1239	306 (24.7)	193 (63.1)	121 (39.5)	755	258 (34.2)	152 (58.9)	113 (43.8)

[1]Speciated to either *S. flexneri* or *S. sonnei*.
