# Supplementary material for: Epidemiology of Shigella infections and diarrhea in the first two years of life using culture-independent diagnostics in 8 low-resource settings
Source: PLoS Negl Trop Dis. 2020 Aug 17;14(8):e0008536. doi: 10.1371/journal.pntd.0008536 (PMC7451981; doi:10.1371/journal.pntd.0008536)
Supplement: S3 Table — (PDF) [file pntd.0008536.s006.pdf]

Table S3. Clinical characteristics of *Shigella*-attributable diarrhea comparing children's first episodes to subsequent episodes among 755 episodes.

Episode characteristic	First episode (N=507) N (%)	Subsequent episode (N=248) N (%)	Risk ratio[1] for characteristic in 1st vs. subsequent episode (95% CI)
Severe (score ≥4)	150 (29.6)	64 (25.8)	1.08 (0.82, 1.41)
Blood	74 (14.6)	37 (14.9)	0.81 (0.55, 1.20)
Fever	164 (32.4)	71 (28.6)	1.05 (0.82, 1.35)
Prolonged (≥7 days)	109 (21.5)	34 (13.7)	1.13 (0.78, 1.64)
Persistent (≥14 days)	22 (4.3)	4 (1.6)	1.75 (0.67, 4.59)
Dehydration	60 (11.8)	16 (6.5)	1.41 (0.84, 2.36)
Vomiting	106 (20.9)	34 (13.7)	1.13 (0.77, 1.66)
High frequency (>6 loose stools in 24 hours)	133 (26.2)	47 (19.0)	1.21 (0.89, 1.63)
Hospitalization	2 (0.4)	0	--

[1]Adjusted for site and age; excludes sites with no *Shigella*-attributable diarrhea episodes with characteristic (Brazil for severe, dehydration, and high frequency; Brazil and South Africa for blood).
