# Supplementary material for: Epidemiology of Shigella infections and diarrhea in the first two years of life using culture-independent diagnostics in 8 low-resource settings
Source: PLoS Negl Trop Dis. 2020 Aug 17;14(8):e0008536. doi: 10.1371/journal.pntd.0008536 (PMC7451981; doi:10.1371/journal.pntd.0008536)
Supplement: S4 Table — (PDF) [file pntd.0008536.s007.pdf]

Table S4. Coinfections during 755 *Shigella*-attributable diarrhea episodes.

Co-pathogen[1]	Episodes with co-pathogen detected[2] N (%)	Episodes with co-pathogen as a second etiology[3] N (%)	Episodes with co-pathogen as primary etiology[4] N (%)
Any	727 (96.3)	289 (38.3)	92 (12.2)
Viruses			
Any	528 (69.9)	197 (26.1)	59 (7.8)
Rotavirus	71 (9.6)	35 (4.7)	15 (2.0)
Norovirus	160 (21.6)	21 (2.8)	4 (0.5)
Adenovirus 40/41	238 (32.0)	77 (10.4)	17 (2.3)
Astrovirus	188 (25.3)	28 (3.8)	11 (1.5)
Sapovirus	229 (30.9)	64 (8.6)	17 (2.3)
Bacteria			
Any	670 (88.7)	106 (14.0)	31 (4.1)
Campylobacter spp.	330 (44.4)	0	0
Campylobacter jejuni/coli	236 (31.7)	16 (2.2)	2 (0.3)
tEPEC	168 (22.6)	2 (0.3)	0
aEPEC	222 (29.8)	1 (0.1)	0
ETEC	401 (53.8)	87 (11.7)	28 (3.8)
EAEC	426 (57.6)	0	0
V. cholerae	4 (0.5)	3 (0.4)	2 (0.3)
Parasites			
Any	426 (56.4)	17 (2.3)	8 (1.1)
Cryptosporidium	114 (15.4)	11 (1.5)	5 (0.7)
Giardia	336 (45.2)	1 (0.1)	0
E. bieneusi	56 (7.5)	0	0
Cyclospora	8 (1.1)	2 (0.3)	1 (0.1)
Isospora	6 (0.8)	2 (0.3)	2 (0.3)
Strongyloides	6 (0.8)	1 (0.1)	0

[1]Pathogen groups include all 29 pathogens tested in MAL-ED; individual pathogen results are listed for pathogens with ≥ 5% prevalence or at least one episode in which it was a second etiology.
[2]Co-pathogen detected at Cq < 35.
[3]Co-pathogen detected and pathogen-specific AFe ≥ 0.5 (i.e. pathogen detected at a quantity high enough to be considered etiologic).
[4]Co-pathogen detected and pathogen-specific AFe was greater than the *Shigella* AFe (i.e. co-pathogen may be primary etiology; subset of episodes from [2] above).
