# Supplementary material for: Epidemiology of Shigella infections and diarrhea in the first two years of life using culture-independent diagnostics in 8 low-resource settings
Source: PLoS Negl Trop Dis. 2020 Aug 17;14(8):e0008536. doi: 10.1371/journal.pntd.0008536 (PMC7451981; doi:10.1371/journal.pntd.0008536)
Supplement: S5 Table — (PDF) [file pntd.0008536.s008.pdf]

Table S5. Clinical characteristics of *Shigella*-attributable diarrhea comparing episodes with *Shigella* as the only etiology identified to episodes with another etiology identified among 755 episodes.

Episode characteristic	*Shigella* only etiology (N=466) N (%)	Viral co-etiology (N=197) N (%)	Risk ratio[1] for co-etiology vs. single etiology (95% CI)	Bacterial co-etiology (N=106) N (%)	Risk ratio[1] for co-etiology vs. single etiology (95% CI)	Parasitic co-etiology[2] (N=17) N (%)
Severe (score ≥4)	130 (27.9)	61 (31.0)	1.19 (0.93, 1.53)	25 (23.6)	0.88 (0.60, 1.29)	6 (35.3)
Blood	80 (17.2)	19 (9.6)	0.60 (0.38, 0.96)	11 (10.4)	0.62 (0.35, 1.11)	2 (11.8)
Fever	151 (32.4)	58 (29.4)	1.05 (0.82, 1.36)	30 (28.3)	1.03 (0.74, 1.44)	6 (35.3)
Prolonged (≥7 days)	91 (19.5)	33 (16.8)	1.15 (0.83, 1.60)	19 (17.9)	1.18 (0.77, 1.80)	5 (29.4)
Persistent (≥14 days)	14 (3.0)	8 (4.1)	--[3]	5 (4.7)	--[3]	0
Dehydration	54 (11.6)	16 (8.1)	1.18 (0.71, 1.97)	6 (5.7)	--[3]	1 (5.9)
Vomiting	78 (16.7)	50 (25.4)	1.79 (1.31, 2.46)	18 (17.0)	1.11 (0.71, 1.74)	1 (5.9)
High frequency (>6 loose stools in 24 hours)	110 (23.6)	47 (23.9)	1.11 (0.84, 1.46)	26 (24.5)	0.99 (0.71, 1.37)	3 (17.7)
Hospitalization	1 (0.2)	1 (0.5)	--[3]	0	--[3]	0

[1]Adjusted for site and age; excludes sites with no *Shigella*-attributable diarrhea episodes with characteristic (Brazil for severe, dehydration, and high frequency; Brazil and South Africa for blood; Brazil, South Africa, and Tanzania for persistent diarrhea).
[2]Risk ratios not estimated due to small numbers of episodes with a parasitic co-etiology.
[3]Risk ratios not estimated due to small numbers of episodes.
