# Supplementary material for: Epidemiology of Shigella infections and diarrhea in the first two years of life using culture-independent diagnostics in 8 low-resource settings
Source: PLoS Negl Trop Dis. 2020 Aug 17;14(8):e0008536. doi: 10.1371/journal.pntd.0008536 (PMC7451981; doi:10.1371/journal.pntd.0008536)
Supplement: S6 Table — (PDF) [file pntd.0008536.s009.pdf]

Table S6. Association between *Shigella* detection and historical monthly average temperature and rainfall from 1982-2012 by site.

Site	Temperature Risk ratio[1] (95% CI)	Rainfall Risk ratio[2] (95% CI)
Dhaka, Bangladesh	1.45 (1.19, 1.78)	1.46 (1.20, 1.77)
Fortaleza, Brazil	0.70 (0.47, 1.04)	1.09 (0.71, 1.68)
Vellore, India	1.32 (1.08, 1.61)	1.11 (0.92, 1.34)
Bhaktapur, Nepal	2.71 (1.96, 3.75)	2.17 (1.67, 2.82)
Loreto, Peru	1.30 (1.07, 1.57)	1.11 (0.91, 1.36)
Naushero Feroze, Pakistan	1.17 (0.88, 1.57)	0.78 (0.61, 1.00)
Venda, South Africa	1.81 (1.33, 2.45)	1.56 (1.23, 1.97)
Haydom, Tanzania	1.92 (1.63, 2.27)	1.72 (1.49, 2.00)

[1]Risk ratio for *Shigella* detection in non-diarrheal stools comparing site-specific high to low monthly temperature (defined by the 90th and 10th percentiles of the monthly average temperature at each site).
[2]Risk ratio for *Shigella* detection in non-diarrheal stools comparing site-specific high to low monthly rainfall (defined by the 90th and 10th percentiles of the monthly average rainfall at each site).
