# Supplementary material for: Epidemiology of Shigella infections and diarrhea in the first two years of life using culture-independent diagnostics in 8 low-resource settings
Source: PLoS Negl Trop Dis. 2020 Aug 17;14(8):e0008536. doi: 10.1371/journal.pntd.0008536 (PMC7451981; doi:10.1371/journal.pntd.0008536)
Supplement: S7 Table — (PDF) [file pntd.0008536.s010.pdf]

Table S7. Site-specific associations between *Shigella* and biomarkers of environmental enteropathy among 19,148 diarrheal and non-diarrheal stools with biomarker measurements.

	Adjusted[1] concentration difference (95% CI)						
Site	Myeloperoxidase (log[ng/mL])	Neopterin (log[nmol/L])	α-1-antitrypsin (log[mg/g])	α-1-acid glycoprotein (mg/dL) [2]	Lactulose:mannitol z-score[3]	Lactulose z-score[3]	Mannitol z-score[3]
Bangladesh	0.32 (0.17, 0.47)	-0.2 (-0.37, -0.02)	0.06 (-0.06, 0.18)	1.82 (-4.39, 8.03)	-0.14 (-0.3, 0.02)	-0.36 (-0.63, -0.08)	-0.08 (-0.29, 0.14)
Brazil	0.70 (0.29, 1.11)	0.5 (0.23, 0.78)	-0.22 (-0.47, 0.03)	14.24 (-0.54, 29.03)	0.17 (-0.21, 0.55)	-0.21 (-0.54, 0.12)	-0.37 (-0.7, -0.05)
India	0.13 (-0.02, 0.27)	-0.11 (-0.24, 0.02)	-0.00 (-0.12, 0.12)	7.12 (-3.71, 17.96)	0.46 (0.27, 0.65)	0.47 (0.15, 0.78)	0.07 (-0.19, 0.34)
Nepal	0.31 (0.11, 0.52)	0.06 (-0.1, 0.22)	-0.11 (-0.3, 0.07)	9.12 (-10.21, 28.46)	-0.21 (-0.69, 0.27)	-0.01 (-0.33, 0.31)	0.16 (-0.27, 0.59)
Peru	0.56 (0.40, 0.71)	-0.16 (-0.3, -0.01)	-0.25 (-0.45, -0.05)	-0.67 (-9.83, 8.49)	0.12 (-0.16, 0.39)	-0.22 (-0.59, 0.14)	-0.35 (-0.63, -0.06)
Pakistan	0.21 (-0.03, 0.46)	0.28 (0.06, 0.5)	-0.05 (-0.27, 0.17)	15.53 (6.26, 24.81)	0.06 (-0.24, 0.37)	-0.12 (-0.62, 0.37)	-0.18 (-0.57, 0.22)
South Africa	0.32 (0.11, 0.52)	0.13 (-0.04, 0.31)	0.1 (-0.09, 0.30)	12.53 (-3.09, 28.15)	0.69 (0.33, 1.05)	0.31 (-0.18, 0.8)	-0.46 (-0.84, -0.07)
Tanzania	0.38 (0.24, 0.51)	0.05 (-0.11, 0.21)	0.11 (-0.01, 0.23)	17.67 (5.78, 29.55)	-0.12 (-0.53, 0.28)	-0.12 (-0.45, 0.21)	-0.04 (-0.32, 0.25)

[1]Adjusted for site, age, sex, and stool consistency.
[2]N=4147 at 7, 15, and 24 months of age; adjusted for site, age, and sex.
[3]Brazil cohort was the internal reference population; N=6110 at 3, 6, 9, and 15 months of age; adjusted for site, age, and sex.
